# Managing venous thrombosis in a pediatric patient with short bowel and congenital nephrotic syndromes: a case report emphasizing rivaroxaban level monitoring

**DOI:** 10.3389/fped.2024.1385065

**Published:** 2024-04-03

**Authors:** Marc Bosch-Schips, Gonzalo Artaza, Carlos Hernández-Mata, Víctor Pérez Beltrán, Vanessa Cabello Ruiz, Pável Olivera Sumire

**Affiliations:** ^1^ Hematology Department, Hospital Universitari Vall d'Hebron, Experimental Hematology, Vall d'Hebron Institute of Oncology (VHIO), Vall d'Hebron Barcelona Hospital Campus, Barcelona, Spain; ^2^ Pediatric Nephrology Department, Hospital Universitari Vall d’Hebron, Barcelona, Spain; ^3^ Pediatric Nutrition and Gastroenterology Department, Hospital Universitari Vall d’Hebron, Barcelona, Spain

**Keywords:** congenital nephrotic syndrome, short bowel syndrome, anticoagulation, direct action anticoagulants, thrombosis

## Abstract

Direct Oral Anticoagulants (DOACs) typically exhibit a predictable pharmacokinetic and pharmacodynamic response at a fixed dose, not necessitating monitoring under standard conditions. Yet, in specific clinical scenarios that can impair it, like Congenital Nephrotic Syndrome (CNS) or Short Bowel Syndrome (SBS) due to absorption issues, anti-thrombin III (AT-III) deficiency and non-selective proteinuria, adjusting the dosage to achieve appropriate plasma concentrations could prove beneficial. We report a 3-month-old female with catheter-related jugular thrombosis affected by CNS concomitant to SBS and failure of both treatments with heparin and warfarin, that was switched to dose-adjusted pediatric rivaroxaban. Rivaroxaban was adjusted to reach peak levels between 189 and 419 ng/ml and the lower trough levels between 6 and 87 ng/ml. Increasing doses were needed due to SBS related malabsorption but a complete permeabilization of the vein was achieved without bleeding complications. The use of anti-Xa adjusted rivaroxaban could be an alternative to improve anticoagulation and secondary thromboprophylaxis in pediatric patients SBS and an option to children with CNS.

## Introduction

Congenital Nephrotic Syndrome (CNS) is characterized by nephrotic range proteinuria, hypoalbuminemia, and edema, often evident *in utero* or within the first 3 months after birth (1). While rare instances may link it to congenital infections or maternal alloimmune diseases, the most severe and widespread cases predominantly stem from a genetic predisposition affecting the structure and function of podocytes, leading to a primary glomerular disorder. Specifically, biallelic pathogenic variants in genes such as NPHS1 or NPHS2 stand out as the most common cause of this syndrome (1–5).

In the trajectory of CNS, most patients progress towards end-stage renal failure within a few years, accompanied by an augmented risk of significant clinical complications such as hemodynamic instability, recurrent infections, growth retardation, and thrombosis (1, 6–8).

Thrombosis prevalence in CNS is estimated to range between 10% and 29% (9–12). Several risk factors, such as the disturbance of the hemostatic balance facilitated by the urinary loss of anticoagulant factors like antithrombin (AT) and plasminogen, dehydration, thrombocytosis, and the utilization of central venous devices contribute to that end (1, 2).

Due to these systemic alterations, optimal anticoagulation management becomes challenging when thrombosis occurs. An advantage in favor of DOACs might be their predictable pharmacokinetic and pharmacodynamic responses, which usually make monitoring unnecessary. However, uncertainties arise when other factors like diminished gastrointestinal drug absorption are involved. In such instances, the potential benefit of frequent drug monitoring to tailor dosing strategies remains uncertain.

## Case report

The case involves a 3-month-old female born prematurely at 33 + 5 weeks to non-consanguineous parents, weighing 1990 g at birth. Post-delivery, she developed a small bowel volvulus linked to bowel malrotation, necessitating surgical intervention that resulted in short bowel syndrome. At birth, clinical indicators suggestive of CNS emerged, including edema, hypoproteinemia, hypoalbuminemia, significant proteinuria with albuminuria (urinary protein/urinary creatinine: 48,908.17 mg/g/urinary albumin/urinary creatinine 36,083.39 mg/g), hypertriglyceridemia, normal renal function (eGFR with Schwartz equation 45.43 ml/min/1.73 m^2^), reduced antithrombin activity (ATa) (21%) and a renal ultrasound without abnormalities. Subsequent analysis via Next-Generation Sequencing focusing on the Finnish variant of CNS revealed compound heterozygosity for the NPHS1 nephrin gene: c.1538T>C p.(Leu513Pro), categorized as likely-pathogenic, and c.3250dup p.(Val1084Glyfs Ter12), categorized as pathogenic.

At 3 months of age, she was transferred to our hospital to assess combined nephrological and nutritional management. She required daily IV albumin infusion and continuous parenteral nutrition, administered through a central venous catheter. Despite prior Low Molecular Weight Heparin (LMWH) thromboprophylaxis, an incidental finding during routine ultrasound indicated a non-occlusive left jugular vein thrombosis associated with the catheter. She had impaired coagulation tests, with partial thromboplastin time (aPTT) ratio 1.6, decreased FIX (33%), FXI (40%) and FXII (13%), a nearly absent ATa (1%) presumably related to the CNS and a negative lupus anticoagulant. Antithrombotic treatment commenced with LMWH, adjusted to achieve an anti-FX activity range between 0.5 and 1 following intravenous AT replacement. However, despite doses of enoxaparin reaching up to 7 mg/kg/12 h, the target was unattainable. Furthermore, AT activity only reached 10%. During this period, the patient presented a new contralateral jugular thrombosis. Anticoagulation therapy transitioned to warfarin at a dose of 0.15 mg/kg, aiming for an international normalized ratio (INR) value between 2.0 and 3.0. Nevertheless, 7 weeks after starting warfarin a new venous thrombosis progression affecting the innominate trunk, left subclavian vein, and axillary vein was diagnosed. Throughout the course of warfarin treatment, the patient's time spent within the therapeutic range was 13%. Complications emerged during her treatment, including bleeding at catheter puncture sites and mild hematuria on two occasions. Additionally, for perioperative anticoagulation during catheter replacements and left nephrectomy (due to the failure of pharmacological induction of renal insufficiency), management with unfractionated heparin (UFH) was necessary. Monitoring was conducted using anti-FXa due to baseline aPTT elevation secondary to acquired FIX and FXI deficiency.

**Figure 1:**
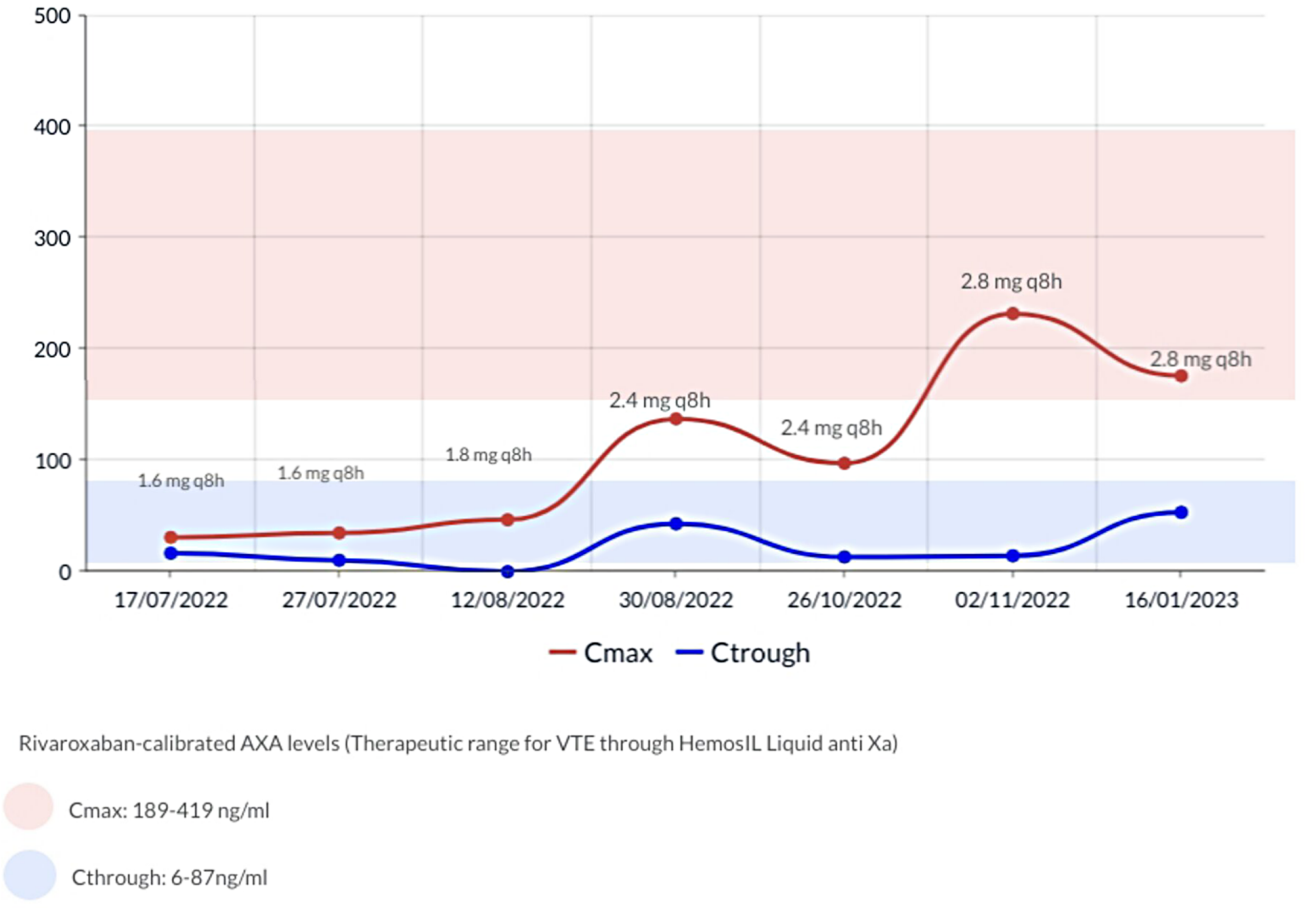
Rivaroxaban-calibrated anti-Xa levels.

The anticoagulation strategy was changed to rivaroxaban post parental authorization. Albeit there are no specific recommendations for monitoring DOAC levels in the pediatric population, concerns regarding potential diminished drug absorption due to Short Bowel Syndrome (SBS) led us to employ a chromogenic Anti-Xa assay, from Technoclone (Vienna-Austria), normalized against a standardized dilutional rivaroxaban concentration curve, to monitor and adjust the dose. Based on prior case reports, our goal was to achieve peak plasma concentrations (2–3 h after the taking) between 189 and 419 ng/ml and trough plasma concentrations (prior to the next dose) 6 and 87 ng/ml. Initially rivaroxaban was started at 1.6 mg/8 h in accordance to the technical sheet for a 5 kg weight and subsequently adjusted as shown in Figure 1.

An ultrasound conducted 12 weeks post initiation of rivaroxaban showed a persistent obliteration of the distal third of the internal left jugular vein with collateral circulation and permeability of all other cervical veins. Upon discharge, at 12 months of age, a new ultrasound revealed complete restoration of the internal left jugular vein's permeability. No bleedings were reported during treatment, and no further hospitalizations were required. Four months later, repeated episodes of regurgitation compromised rivaroxaban intake, and the patient was switched back to enoxaparin at a dose of 2.5 mg/kg/12 h, controlled by anti-FXa in the range of intermediate dosages 0.4–0.5 without further complications.

At 20 months old, kidney transplantation was performed. Two weeks later, considering the history of thrombosis and the persistent need of the CVC for parenteral nutrition, rivaroxaban was reintroduced with adequate oral tolerance for secondary prevention of thrombosis.

## Discussion

Thrombotic complications significantly impact both short- and long-term prognosis in patients with CNS. However, the optimal strategy for treating and preventing thrombosis, particularly in children under 2 years old, remains a topic of debate. Physiological changes resulting from CNS pose theoretical and practical challenges in managing all available anticoagulants.

A published series involving 8 patients with CNS highlighted the necessity for higher heparin doses for venous thromboprophylaxis aiming a target Anti-FXa between 0.2 and 0.4 IU/ml (7). Despite several dosage adjustments, up to a maximum of 7.44 mg/kg/day in one of the cases, 50% of the patients required a switch to warfarin. Among these cases, only 2 patients reached therapeutic INR levels after 6 and 11 weeks of adjustments, while the remaining 2 did not reach therapeutic INR, with one patient undergoing 1 year of dosage adjustments.

Recent evidence showcasing the efficacy and safety of rivaroxaban and dabigatran as anticoagulants in the pediatric population broadens the therapeutic options for this patient group (13–15). These medications offer advantages over other anticoagulants owing to their oral administration, rapid onset of action, independence from AT activity, and availability in specific pediatric formulations. However, controversy persists regarding the efficacy and safety of these drugs in cases of Nephrotic Syndrome (NS) due to their high protein binding affinity (rivaroxaban 90%–95% and dabigatran 34%–35%), in the face of proteinuria, alterations in volume distribution or changes in plasma concentrations of coagulation factors (16).

A published series encompassed 4 pediatric patients (>16 years) with NS and thrombosis who were treated with rivaroxaban. The median recorded rivaroxaban level stood at 248 ng/ml (160–342), but no specific adjustments were reported (17). Also, no treatment failures or major bleeding complications were identified. The case of a 13-year-old patient with NS who presented PE and DVT, effectively managed with rivaroxaban 20 mg/24 h due to the difficulty of maintaining anticoagulation with AT and UFH infusions, was also recently published (18). Random rivaroxaban levels (12 h) and anti-Xa activity were measured at 99 ng/ml and 0.14 IU/ml respectively. However, no specific adjustments were detailed in this instance.

Managing anticoagulation can become significantly more complex when dealing with a concurrent malabsorptive condition. Rivaroxaban, being absorbed in the proximal part of the digestive tract, might potentially serve as an alternative treatment regimen if this portion remains sufficiently preserved. There is substantial data investigating bioavailability and absorption of anticoagulants in individuals following bariatric procedures, but there is a notable absence of data concerning the pharmacokinetics or pharmacodynamics of rivaroxaban in pediatric patients with SBS (19, 20).

In our case, the aforementioned considerations prompted us to monitor rivaroxaban levels to ensure optimal anticoagulation efficacy. To our knowledge, this is the first case report of a CNS and SBS patient under 2 years old, receiving treatment and secondary prophylaxis with rivaroxaban, wherein plasma levels where actively utilized to adjust drug dosage. In addition, it illustrates the need for higher doses per weight to reach target plasmatic levels due to SBS, distribution volume, renal function, metabolism, and patient growth. Importantly, the absence of hemorrhagic complications also suggests a favorable safety profile.

## Conclusion

The use of anti-Xa adjusted rivaroxaban stands as a potentially safe alternative to enhance anticoagulation and secondary thromboprophylaxis in pediatric patients with malabsorptive conditions that could compromise pharmacodynamics predictability. Moreover, its mechanism of action independent from AT could be advantageous in CNS patients. Prospective studies are essential to further explore the efficacy and safety of DOACs in these clinical settings.

## Data Availability

The original contributions presented in the study are included in the article/Supplementary Material, further inquiries can be directed to the corresponding author.
